# The long term effect of a multidimensional comprehensive cognitive intervention training program in cognitive impairments: a longitudinal study in China

**DOI:** 10.1002/alz.090090

**Published:** 2025-01-09

**Authors:** Feng Yang, Yatian Li, Zhixing Zhou, Ruihan Wang, Hanlin Cai, Huanhuan Xia, Qin Chen

**Affiliations:** ^1^ West China Hospital of Sichuan University, Chengdu China; ^2^ Shanghai Bestcovered Limited, Shanghai China

## Abstract

**Background:**

Older adults with cognitive impairments will benefit from multicomponent interventions include cognitive training, exercise, and lifestyle modifications. Many digital therapeutic products predominantly focus on computerized cognitive training, lacking effective approaches to other crucial interventions. This study aimed to investigate the long term effects of multidimensional comprehensive cognitive intervention training program – Brain and Body Rehab Training (BBRT), which integrates multidomain cognitive training with physical‐cognitive training and multidimensional lifestyle interventions on cognitive performance in participants with cognitive impairment after a 8‐month follow‐up.

**Method:**

All participants (n = 351) were allocated into the cognitive unimpaired (CU) group and cognitive impaired (CI) group. Participants were evaluated by cognitive and performance test batteries before, 4 months and 8 months after the interventions. In both groups, an individualized training program is assigned consisting of completing four to five daily tasks, including cognitive training, physical‐cognitive training, lifestyle interventions, chronic disease/diet/sleep/emotion management, and traditional Chinese medicine non‐pharmacological interventions among others. The intervention duration ranges 15‐25 minutes per day, and task difficulty is dynamically adjusted based on individual task performance and periodic cognitive assessments. Participants were asked to take the intervention 3‐4 times per week for 8 months. The statistic analysis were using mixed‐effect regression model.

**Results:**

After the 4 months intervention, a significant improvement on the cognitive performance was observed in the CI group compared to the CU group (p<0.001), and the improvement persistent after 8 month follow‐up (p<0.001). The change of cognitive performance were associated with frequency of training repetition and whole time of the intervention.

**Conclusions:**

The BBRT program significantly improved the cognitive function in cognitively impaired participants after 4 months training and had a persistent effect after 8 months. The convenient home‐based multidimensional interventions provides an long term effects for the cognitive impaired participants.

